# Aluminum Melt Filtration with Carbon Bonded Alumina Filters

**DOI:** 10.3390/ma13183962

**Published:** 2020-09-07

**Authors:** Claudia Voigt, Jana Hubálková, Tilo Zienert, Beate Fankhänel, Michael Stelter, Alexandros Charitos, Christos G. Aneziris

**Affiliations:** 1 Institute of Ceramic, Glass and Construction Materials, Technische Universität Bergakademie Freiberg, Agricolastraße 17, 09599 Freiberg, Germany; jana.hubalkova@ikgb.tu-freiberg.de (J.H.); tilo.zienert@ikgb.tu-freiberg.de (T.Z.); aneziris@ikgb.tu-freiberg.de (C.G.A.); 2 Institute for Nonferrous Metallurgy and Purest Materials, Technische Universität Bergakademie Freiberg, Leipziger Straße 34, 09599 Freiberg, Germany; beate.fankhaenel@inemet.tu-freiberg.de (B.F.); stelter@inemet.tu-freiberg.de (M.S.); alexandros.charitos@inemet.tu-freiberg.de (A.C.)

**Keywords:** filtration, aluminum melt, ceramic foam filter, Al_2_O_3_-C, thermodynamics

## Abstract

The wetting behavior was measured for Al_2_O_3_-C in contact with AlSi7Mg with a conventional sessile drop test (vacuum, 950 °C and 180 min) and a sessile drop test with a capillary purification unit (vacuum, 730 °C and 30 min). The conventional test yielded contact angles of around 92°, whereas the sessile drop measurement with capillary purification showed a strongly non-wetting behavior with a determined apparent contact angle of the rolling drop of 157°. Filtration tests, which were repeated twice, showed that the Al_2_O_3_-C filter possessed a better filtration behavior than the Al_2_O_3_ reference filter. For both filtration trials, the PoDFA (porous disc filtration analysis) index of the Al_2_O_3_-C filter sample was equal to half of the PoDFA index of the Al_2_O_3_ reference filter sample, indicating a significantly improved filtration performance when using Al_2_O_3_-C filter. Notable is the observation of a newly formed layer between the aluminum and the Al_2_O_3_-C coating. The layer possessed a thickness between 10 µm up to 50 µm and consisted of Al, C, and O, however, with different ratios than the original Al_2_O_3_-C coating. Thermodynamic calculations based on parameters of the wetting and filtration trials underline the possible formation of an Al_4_O_4_C-layer.

## 1. Introduction

The filtration of metal melt with ceramic foam filters is the state of the art since the 1960s. Ceramic foam filters used for the filtration of aluminum and aluminum alloys are typically made of alumina (Al_2_O_3_) or silicon carbide (SiC) [1]. In comparison, for the filtration of steel melts, ceramic foam filters predominantly based on zirconia (ZrO_2_) or carbon bonded alumina (Al_2_O_3_-C) are industrially applied [2]. Al_2_O_3_-C is a refractory material with a low thermal expansion, negligible sinter shrinkage and improved slagging resistance caused by the low wetting (high contact angle) between carbon-bonded alumina and metallic melts. The main drawback of Al_2_O_3_-C is the low resistance against oxidation at high temperatures.

According to the filtration trials conducted by Voigt et al., an increasing contact angle induces an enhanced filtration behavior [3]. Based on this assumption, the low wetting between the Al_2_O_3_-C and metal melts might be beneficial in terms of high filtration efficiency. Al_2_O_3_-C materials are comprised of alumina, carbon fillers (e.g., carbon black and graphite) and carbon binders (e.g., pitch, tar, or phenolic resin), as well as appropriate additives [4]. The replica technique, based on coating of a polymeric template with a ceramic slurry followed by multi-step thermal treatment (drying, burning out of the polymer, sintering or coking), has been proved suitable for the production of ceramic foam filters based on Al_2_O_3_-C [5,6].

The aim of this study was to correlate the results of industrially conducted aluminum filtration trials using Al_2_O_3_-C ceramic foam filters with the wetting behavior of Al_2_O_3_-C substrates by aluminum.

For the determination of the wetting behavior and contact angle between solids and metal melts, the sessile drop technique is often used due to the relatively simple experimental setup. The testing procedure comprises the placing of a piece of metal on the substrate and subsequent heating in a furnace. During the heating and dwelling time, the metal drop shape is recorded and analyzed [7].

The determination of the contact angle of aluminum melt is influenced by numerous factors, on the part of aluminum (amount of impurities and alloying elements), testing conditions (atmosphere, temperature and time) as well as the part of the substrate (surface roughness, phase composition, crystal orientation and chemical heterogeneity) [8,9,10]. A strong impact on the measured contact angle is given by the affinity of aluminum to oxygen and, therefore, the formation of an alumina skin.

Even nano-scaled oxide skins cause a systematically higher and nearly constant contact angle. To remove the oxide skin, temperatures higher than 950 °C, adequate vacuum and relatively long dwelling times are necessary [11]. Such conditions entail the application of temperature being approx. 200 °C higher than the conventional aluminum filtration temperature (between 650 °C and 750 °C), of different atmosphere (filtration trials are conducted under air) and of longer dwelling times. The conditions equivalent to the real aluminum filtration are feasible only using a sessile drop facility equipped with a capillary purification unit, where the aluminum metal is melted separately from the substrate. After melting, an oxide skin-free aluminum is dropped onto the substrate, since the oxide skin remains in the dropping unit, which allows the application of temperature lower than 950 °C [10].

The focus of the paper lies on the filtration of aluminum alloy melt. According to Damoah et al. [12], the filtration depends on multi-facetted parameters such as metal melt parameters (temperature, viscosity and composition), inclusion parameters (structure, size, number and chemistry), process parameters (melt velocity, casting design, melt preparation), and filter parameters (geometry, functional pore size, relative porosity, wetting behavior, chemistry). Despite the application of ceramic foam filters since the 1970s, there is little information about the influence of the filter chemistry on the filtration performance. Görner et al. [13] applied a filter with an AlF_3_ surface for filtering of Na and Mg from aluminum melts and achieved a Na removal of 80% to 91%. Zhou et al. [14] tested an enamel coating and observed an improvement of tensile strain and a decrease of inclusions with a size of 6 µm. A NaBr coating was used by Luyten et al. [15] for the filtration of intermetallic phases with negative outcomes since large needle-shaped intermetallic phases blocked the coated filter and resulted in a cold run. The aforementioned papers investigated coatings not being used in the industry. In contrast, Syvertsen et al. [16] compared the filtration behavior of two commercially available and industrial used filters types Al_2_O_3_ and SiC both in laboratory and industrial scale. The LiMCA measurements yielded higher filtration efficiencies for filters made of SiC. Voigt et al. [17] investigated filters made of the refractory materials Al_2_O_3_, MgAl_2_O_4_, 3Al_2_O_3_·2SiO_2,_ and TiO_2_. The 3Al_2_O_3_·2SiO_2_ showed the best filtration efficiencies for inclusions <120 µm. As the 3Al_2_O_3_·2SiO_2_ filters also yielded the highest surface roughness, a new issue concerning the role of the filter roughness raised. Further investigations showed a significant, directly proportional impact of the filter roughness on the filtration efficiency [18].

For the evaluation of the filtration behavior, the melt quality regarding the amount of non-metallic inclusions has to be evaluated. The PoDFA (porous disc filtration analysis) technique is often used for the determination of the quantity and nature of the non-metallic inclusions by pressing the aluminum melt through a fine-mesh sieve to collect the non-metallic inclusions of the investigated aluminum alloy melt (up to 3 kg) in the filter cake and to enhance the concentration of the inclusions. After solidification of the aluminum within the filter, the filter including filter cake is cut out. A cross section is polished and analyzed by light microscopy. For reasons of comparability, it is necessary to entirely distinguish between the different inclusion phases (according to the color, morphology and size of the inclusions) and therefore apply a precise analysis specification describing all known characteristics. For the quantitative evaluation, a grid for the determination of the inclusion area is used so that the different kinds of inclusions can be quoted in mm^2^ inclusions per kilogram of analyzed aluminum. The amount and size of the oxide films is roughly estimated. Furthermore, it should be kept in mind that there are two kinds of PoDFA analysis, hot and cold. The hot PoDFA operates with aluminum melt samples taken directly from the process, whereas a cold PoDFA uses a solidified aluminum sample that has to be melted just before the test. The renewed melting needed for cold PoDFA might initiate the formation of further inclusions and oxide films, negatively affecting the analysis results [19,20]. 

## 2. Materials and Methods

### 2.1. Preparation of the Ceramic Foams and Substrates

For the investigation of the wetting and filtration behavior, two different sample geometries were necessary. The measurement of the contact angle was conducted using bulk substrates in the form of tablets having a diameter of 10 mm and a height of 6 mm, whereas for the filtration trials, ceramic foam filters in the form of cuboids having an edge length of 50 mm and a height of 22 mm with 20 ppi (pores per inch) were applied. The results of the filtration and the wetting measurements were compared to pure Al_2_O_3_ filters and pure Al_2_O_3_ substrates used as reference materials. In order to be directly able to compare the filtration behavior of Al_2_O_3_-C filter with the reference, an equivalent functional pore size distribution was required. The real challenge is the sinter shrinkage of ceramic foam filters made of pure alumina during the thermal treatment. Contrary to pure alumina, Al_2_O_3_-C exhibits no significant shrinkage. For all investigations within this study, Al_2_O_3_ skeleton filters were used and coated with Al_2_O_3_ slurry (see Table 1) or Al_2_O_3_-C slurry (see Table 2) to prevent the differences in the functional pore size distribution caused by different shrinkage behavior.

The coatings were applied using a combined dip-spin technique where the Al_2_O_3_ skeleton was immersed completely in the slurry followed by a centrifugation step for the removal of excess slurry. The applied thermal treatment conditions are presented in Table 1 and Table 2.

The mechanical stability of the Al_2_O_3_-C coating on the Al_2_O_3_ skeleton filter was tested with a moderate impingement test comprising of the passage of 3 kg AlSi7Mg0.3 melt (Trimet Aluminium AG, Essen, Germany) through the filter. The drop height of the aluminum melt from the crucible to the filter was approximately 30 cm and the melt temperature was approximately 730 °C. The microstructure of the Al_2_O_3_-C-coated filter after the impingement test was evaluated by means of a scanning electron microscope XL 30 SEM (Philips, Eindhoven, Germany).

The substrates for the sessile drop tests were prepared by uniaxial pressing of the dried and crushed slurries (Al_2_O_3_ and Al_2_O_3_-C) as used for the coating of the ceramic foam filters. During the conventional sessile drop test, a coked and polished substrate surface was used. For the sessile drop test with capillary purification, a comparable surface roughness and quality for filters should be achieved. Thus, the uniaxially pressed substrates were dip coated with the coating slurry of the respective compositions. The thermal treatment of the substrates was performed in the same way as for the ceramic foam filters.

### 2.2. Sessile Drop Tests

Two types of sessile drop tests were conducted

- conventional sessile drop test and
- sessile drop test with capillary purification unit.

The conventional sessile drop test was performed at a high-temperature tube furnace with a high vacuum and an inert gas system (Carbolite Gero, Neuhausen, Germany) at the Institute for Nonferrous Metallurgy and Purest Materials (TU Bergakademie Freiberg, Freiberg, Germany). The aluminum alloy AlSi7Mg (Trimet Aluminium, Essen, Germany) was cut to masses between 90 mg to 95 mg immediately before the trial for attaining a thin oxide layer [21], and thereafter placed on the substrates (Al_2_O_3_-C 800 °C and Al_2_O_3_ reference) at room temperature and positioned in the furnace. Before starting the heating procedure with 350 °C/h to the temperature of 950 °C, the furnace was evacuated to reach a pressure of *p* ≤ 1.5 × 10^−5^ mbar. After a dwell time of 180 min, the pressure of *p* < (2.8 ± 0.4) × 10^−5^ mbar was achieved. Three measurements were conducted for the Al_2_O_3_-C substrates.

For the evaluation of the contact angle *θ_cal_*, following the equation valid for small droplets (*m* < 100 mg), Equation (1):

(1) $$\theta_{cal}=2\arctan(2h/d)$$

was used [7]. The height *h* and the diameter *d* of the aluminum droplet were read off from the digital images recorded with a digital camera (The Imaging Source, Bremen, Deutschland).

The sessile drop tests with capillary purification unit were carried out at the Foundry Research Institute (Krakow, Poland) [22]. The aluminum alloy AlSi7Mg (Trimet Aluminium AG, Essen, Germany) was melted in a graphite syringe separately from the Al_2_O_3_-C substrate. At a temperature of 730 °C and a pressure *p* < 1 × 10^−5^ mbar, an oxide-free droplet was placed on the substrate. The shape of the drop was recorded by high-resolution CCD camera with a rate of 100 images per minute.

### 2.3. Filtration Trials

**Figure 1:**
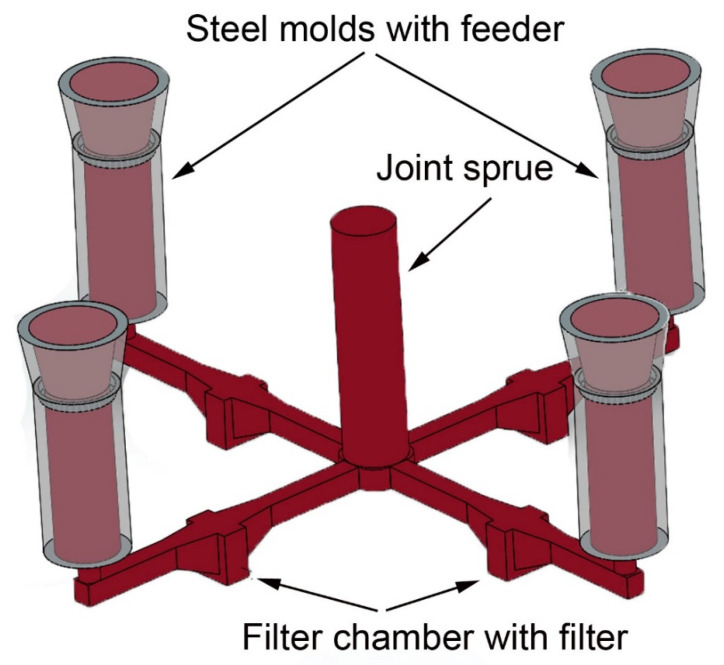
Casting system for the filtration trials with sand mold.

The filtration trials were conducted at the metal foundry Georg Herrmann Metallgiesserei (Muldenhütten, Germany) with the aluminum alloy AlSi7Mg (EN AC-42100 from Rheinfelden Alloys (Rheinfelden, Germany), whereby the aluminum melt comprised 50% ingots and 50% scrap (recycled aluminum consisting of solidified feeders and runners) for the introduction of non-metallic inclusions. The quantity of 300 kg of aluminum alloy AlSi7Mg was electrically heated and skimmed after melting. The used sand mold consisted of a joint sprue, horizontal runners with filter chamber and vertical steel molds (coated with zircon coating), see Figure 1.

The joint sprue should equally distribute the incoming particle load and allow to compare the filtration results of four different filters in the same sand mold, and thus, with comparable melt quality. The investigated ceramic foam filters were tested in two different filtration trials. Filtration trial 1 involved:

- Al_2_O_3_ reference filter
- Al_2_O_3_-C filter (800 °C)

The filtration trial 2 with the following filters was conducted for repeatability reasons:

- Al_2_O_3_ reference filter
- Al_2_O_3_ rough filter
- Al_2_O_3_-C filter (800 °C)
- Al_2_O_3_-C filter (1400 °C)

After the solidification of the aluminum alloy, the casting was detached from the sand and the steel mold. The filters were cut out and embedded with epoxy resin, ground, polished and analyzed with the XL 30 SEM (Philips, Eindhoven, Germany) equipped with an energy-dispersive X-ray spectroscopy device (Phoenix, Weiterstadt, Germany). The aluminum in the steel mold was separated from the feeder and then analyzed regarding non-metallic inclusions with the help of a cold PoDFA analysis performed by HOESCH Metallurgical Service (Niederzier, Germany).

### 2.4. Thermodynamic Calculations

Thermodynamic calculations of chemical reactions between the aluminum melt and the investigated substrate under different experimental conditions were done using the software ThermoCalc [23] based on a self-developed database of the Al-Si-Mg-O-C system [24,25,26].

For the calculations, the interface systems were defined as follows. The interface melt/filter is simulated as a mole fraction of 0.5 atoms of melt and a mole fraction of 0.5 filter atoms. To study the influence of atmosphere, the system was expanded to the respective mole fractions of 0.25 melt atoms, 0.25 atmosphere atoms and 0.5 filter atoms. As nitrogen is not included in our thermodynamic description, air was simulated as a mixture of Ar and O with mole fractions of 0.79 and 0.21, respectively. The composition of the melt is 92.508 at.% Al, 6.813 at.% Si and 0.679 at.% Mg. The Al_2_O_3_-30 wt.%-C filter corresponds to a composition of 23.153 at.% Al, 34.730 at.% O and 42.117 at.% C.

## 3. Results

### 3.1. Preparation of the Ceramic Foams and Substrates 

SEM investigations of the Al_2_O_3_-C-coated filter after the moderate impingement test showed no extensive spalling, peeling or erosion of the coating. The Al_2_O_3_-C coating at the Al_2_O_3_ skeleton proved to be usable.

### 3.2. Sessile Drop Tests

**Figure 2:**
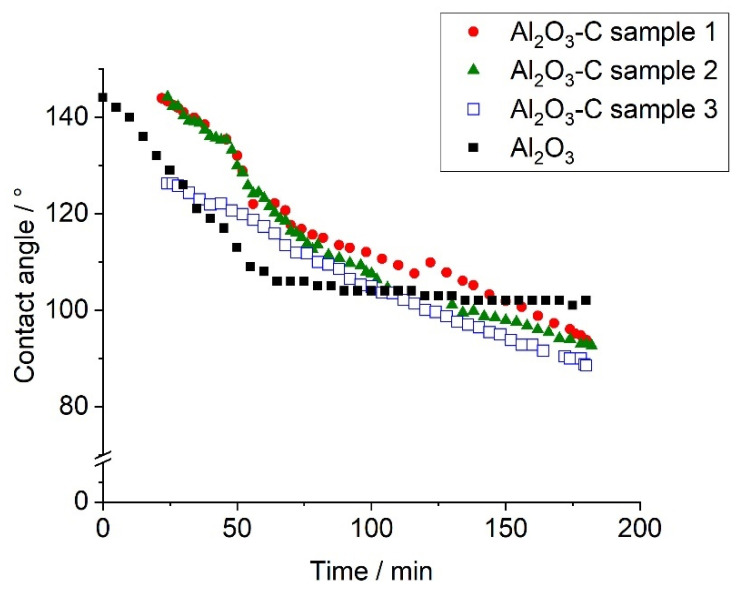
Time dependency of the contact angle determined by sessile drop measurement.

The contact angles measured with the conventional sessile drop technique showed a typical curve progression comprising a decrease of the determined contact angle, see Figure 2.

The contact angle of pure Al_2_O_3_ was above 140° at the beginning of the dwell time at 950 °C and decreased systematically during the dwelling time down to an almost stable contact angle of 100°. The initial decrease of the contact angle indicated that the decomposition of the oxide skin on the aluminum alloy finalized when reaching a stable contact angle.

**Figure 3:**
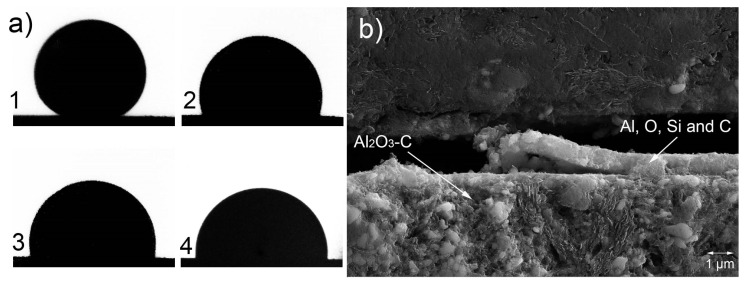
Conventional sessile drop experiment at 950 °C. (**a**) Photo sequence of the sessile drop experiment, (**b**) SEM micrograph of the Al_2_O_3_-C sample after conventional sessile drop test with AlSi7Mg with interfacial layer without AlSi7Mg drop due missing attachment between drop and substrate.

The Al_2_O_3_-C-coated substrates showed different progress with an almost linear decrease of the contact angle over the whole dwelling time without reaching a stable contact angle. The non-achievement of a stable contact angle for the Al_2_O_3_-C substrates can be caused by a reaction between the substrate and the aluminum melt, a strong evaporation of the aluminum melt or a diffusion of the aluminum into the Al_2_O_3_-C substrate. Investigations of the samples after the sessile-drop measurements showed that a reaction between the AlSi7Mg melt and the Al_2_O_3_-C substrate had taken place. No evidence of infiltration of the substrate within the metal could be found, see Figure 3.

On the contrary, the drops could be easily detached from the substrate, with reaction layers visible to the naked eye. Subsequent scanning electron microscopic and energy dispersive X-ray spectroscopy investigations revealed an oxidic layer consisting of Al, O, Si, and C on top of the Al_2_O_3_-C substrate at the former interface and at the bottom of the droplets, Figure 3.

In order to estimate the repeatability of the sessile drop measurements regarding carbon-containing materials, three Al_2_O_3_-C-coated substrates with the same composition and manufactured under the same conditions and coked at 800 °C (designated as Al_2_O_3_-C sample 1 to Al_2_O_3_-C sample 3) were measured. The three performed measurements revealed small differences in the initial stage but at dwell times larger than 150 min, the curve progresses became comparable with total differences of only 6° (contact angles of 89°, 93° and 95° after 180 min). Contact angles lower than 90° [7] indicated a good wetting behavior contradicting the statement of the good slagging resistance due to a poor wetting. Fankhänel et al. [11] proved the occurrence of chemical reactions between several oxide ceramics and an AlSi7Mg alloy during the sessile drop measurements at 950 °C contrary to the measurements performed at 730 °C [3]. The substrate based on MgAl_2_O_4_ showed an impoverishment of Mg after the sessile drop measurements at 950 °C, whereas the same substrate featured no chemical changes after the sessile drop measurements at 730 °C [3].

**Figure 4:**
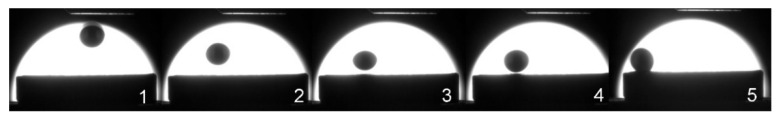
Photo sequence of the sessile drop experiment on Al_2_O_3_-C substrate with capillary purification of AlSi7Mg alloy—Measurement 1.

Therefore, sessile drop measurements with a capillary purification unit at 730 °C were conducted additionally. The greatest challenge of this measurement was the placing of the aluminum droplet at the substrate as the aluminum droplet tended to provide a springing (jumping) movement at the substrate surface before rolling away, see Figure 4.

The repulsive interaction indicates a strong non-wetting behavior between Al_2_O_3_-C and AlSi7Mg. Preceding sessile drop measurements with substrates made of Al_2_O_3_, MgAl_2_O_4_, 3Al_2_O_3_·2SiO_2_, and TiO_2_ did not show such repulsive behavior [3].

**Figure 5:**
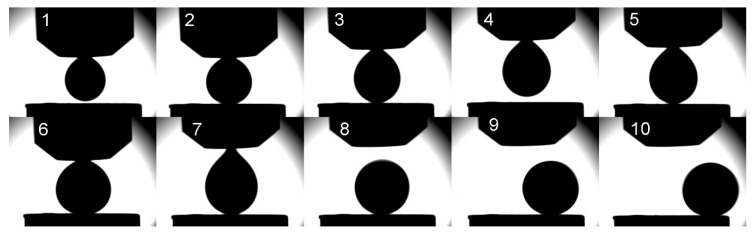
Sequence of photos of the sessile drop experiment on Al_2_O_3_-C substrate with capillary purification of AlSi7Mg alloy—Measurement 2.

A further sessile drop measurement with a modified capillary purification unit, which allows to squeeze and suck the aluminum melt, was carried out. By means of the modified unit, it was possible to lift and lower the syringe. Thus, better positioning of the aluminum droplet without fall and impingement was applicable. Despite this achievement, the aluminum droplet rolled again out of the Al_2_O_3_-C substrate, see Figure 5.

Measurement 2 verified the repulsive interactions and hence a distinct non-wetting behavior between the AlSi7Mg droplet and the Al_2_O_3_-C substrate. The determination of the apparent contact angle between Al_2_O_3_-C and the rolling AlSi7Mg droplet (three last images of Figure 5) yielded a value of (157° ± 1°), whereas the contact angle between Al_2_O_3_ and AlSi7Mg was measured to be 108° [3]. 

The difference between the conventional sessile drop and the sessile drop with capillary purification clearly showed the importance of the testing conditions for determination of the contact angle by aluminum melt. Sessile drop measurements at higher temperatures might enhance reactions not occurring within an industrial aluminum filtration setup.

### 3.3. Filtration Trials

Two different filtrations trials were conducted to test the repeatability of the filtration trials. The filtrations trials were video recorded and showed an equal aluminum filling within 16 s, indicating comparable filter flow rates.

The PoDFA analysis yields the amount of inclusions (area of inclusions in mm^2^ per kilogram analyzed aluminum) detected in the aluminum melt, i.e., the lower the amount of non-metallic inclusions in the aluminum sample, the lower the PoDFA index. The PoDFA index is the sum of the amounts of detected inclusions. Within filtration trial 1, Al_2_O_3_ reference filter and Al_2_O_3_-C filter (800 °C) were tested. Table 3 shows that Al_2_O_3_ films, carbides, magnesium oxide, spinel, refractory material, iron and manganese oxides, as well as grain refiner were detected in the castings.

The PoDFA index of the Al_2_O_3_-C filter sample was equal to half of the PoDFA index of the Al_2_O_3_ reference filter sample, indicating a significantly improved filtration when using Al_2_O_3_-C filter, which mainly resulted from the reduction of spinel inclusions, see Table 3.

Within the scope of filtration trial 2, Al_2_O_3_ reference filter, Al_2_O_3_ rough filter, Al_2_O_3_-C filter (800 °C), and Al_2_O_3_-C filter (1400 °C) were tested. Due to a cold run of the Al_2_O_3_ reference filter (blockade of the filter) during filtration trial 2, in the following, the Al_2_O_3_ rough filter will be used as a reference filter. The substitution of the Al_2_O_3_ reference filter through the Al_2_O_3_ rough filter is justifiable given the fact that previous filtration trials demonstrated better filtration efficiencies for the Al_2_O_3_ rough filter in comparison with the Al_2_O_3_ reference filter [18]. 

The PoDFA results of filtration trial 2 exhibited inclusions of the same nature as for filtration trial 1, see Table 4, as expected due to the application of the same aluminum alloy at the same conditions.

The PoDFA index of the filtered aluminum showed the lowest values for the Al_2_O_3_-C (800 °C) filter followed by the Al_2_O_3_-C (1400 °C) filter. As already mentioned, the temperatures noted in Table 2 refer to the coking temperature of the Al_2_O_3_-C coating. Inversely, the Al_2_O_3_ rough filter possessed the highest PoDFA index, indicating the lowest filtration performance within filtration trial 2.

Both Filtration trials were performed with the same amount of AlSi7Mg scrap material (50%) for the addition of non-metallic inclusions. Due to differences in the production of the metal foundry Georg Herrmann Metallgiesserei (Muldenhütten, Germany), the scrap material introduced different amounts of non-metallic inclusions. Due to the strong influence of the amount of non-metallic inclusions on the filtration rate, there are differences in the PoDFA index between filtration trial 1 and 2. Nevertheless, filtration trials 1 and 2 yielded the same result: the Al_2_O_3_-C filter possessed a better filtration than the Al_2_O_3_ reference filter. The difference between the Al_2_O_3_-C 800 °C and Al_2_O_3_-C 1400 °C filter is not explainable at the moment. Possible reasons are differences in the roughness, the specific surface or the level of graphitization of the Al_2_O_3_-C.

**Figure 6:**
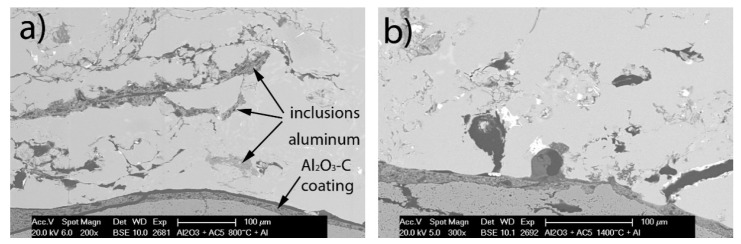
SEM micrographs of the (**a**) Al_2_O_3_-C 800 °C and (**b**) Al_2_O_3_-C 1400 °C after casting of AlSi7Mg with non-metallic inclusions.

SEM micrographs of the filters with the solidified aluminum in BSE mode showed clearly the presence of non-metallic inclusions, see Figure 6. 

**Figure 7:**
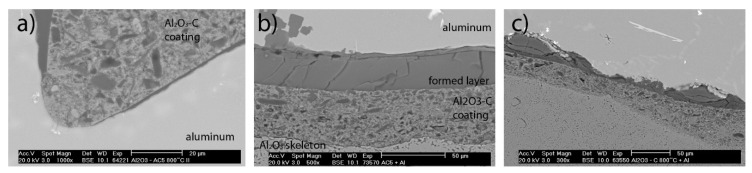
SEM micrographs of the (**a**) Al_2_O_3_-C 800 °C, (**b**) Al_2_O_3_-C 1400 °C and (**c**) Al_2_O_3_ rough filters after casting of AlSi7Mg with non-metallic inclusion.

Notable is the observation of a newly formed boundary layer between the aluminum and the Al_2_O_3_-C coating, see Figure 7b,c.

It should be mentioned that such layers were not visible in each trial. In total, eight casting trials with Al_2_O_3_-C filters were conducted and four of them showed a clear formation of the boundary layer. The thickness varied between 10 and 50 µm. Figure 7a shows a filter without any boundary layer, whereas Figure 7b,c presents such a newly formed boundary, exhibiting different characteristics. The factors and the respective mechanism of boundary layer formation are unclear at the time of writing.

**Figure 8:**
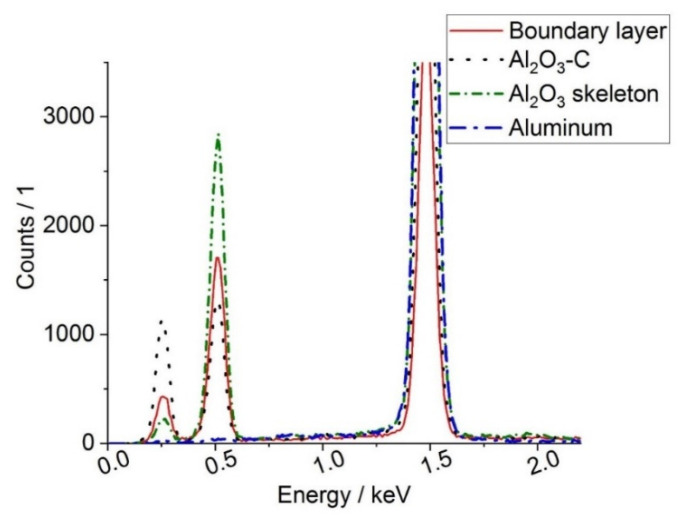
EDX analyses of the newly formed boundary layer (red), Al_2_O_3_-C coating (black), Al_2_O_3_ skeleton filter (green), and solidified aluminum (blue)**.**

The newly formed boundary layer in Figure 7b seemed to be very homogeneous. EDX analyses (Figure 8) of the Al_2_O_3_-C coating and the newly formed layer exhibited the same elements C, O, and Al, but in different ratios.

The newly formed layer features less C, but more O than the Al_2_O_3_-C coating. An investigation of the newly formed boundary layers by means of EBSD revealed no electron backscatter patterns. The missing EBSD pattern might indicate the amorphous character of the newly formed layer. As the newly formed boundary layer showed characteristics of epoxy resin, epoxy resin free samples were tested showing the boundary layer as well. So embedding in epoxy resin can be excluded as reason for the boundary layer.

### 3.4. Thermodynamic Calculations

The results of thermodynamic calculations for the melt/filter interface at different conditions can be written as follows. At atmospheric pressure and a temperature of 740 °C, the liquid partially reacts with the filter as, Equation (2):

(2) $$\begin{array}{cc} 0.5\;L\left(\mathrm{Al},\mathrm{Si},\mathrm{Mg}\right) & +0.5\;\mathrm{filter}\\ & \to 3.89\times 10^{-1}{\;\mathrm{Al}}_{8}{\mathrm{SiC}}_{7}+3.63\times 10^{-1}{\mathrm{Al}}_{4}O_{4}C+2.26\times 10^{-1}L\left(\mathrm{Al},\mathrm{Si},\mathrm{Mg}\right)\\ & +2.17\times 10^{-2}\mathrm{spinel} \end{array}$$

Whereas, under experimental vacuum conditions (*p* = 1 × 10^−5^ mbar) and a temperature of 730 °C, the formation of gas can be expected, Equation (3):

(3) $$\begin{array}{cc} 0.5\;L\left(\mathrm{Al},\mathrm{Si},\mathrm{Mg}\right) & +0.5\;\mathrm{filter}\\ & \to 3.91\times 10^{-1}{\;\mathrm{Al}}_{4}O_{4}C+3.82\times 10^{-1}{\;\mathrm{Al}}_{8}{\mathrm{SiC}}_{7}+2.24\times 10^{-1}L\left(\mathrm{Al},\mathrm{Si},\mathrm{Mg}\right)\\ & +3.53\times 10^{-3}\mathrm{gas}\left(\mathrm{Mg},\mathrm{Al},{\mathrm{Al}}_{2}O\right) \end{array}$$

With increasing temperature, the formation of gas is preferred under experimental vacuum (*p* = 1.5 × 10^−5^ mbar) and a temperature of 950 °C as, Equation (4):

(4) $$\begin{array}{cc} 0.5\;L\left(\mathrm{Al},\mathrm{Si},\mathrm{Mg}\right) & +0.5\;\mathrm{filter}\\ & \to 4.06\times 10^{-1}\;\mathrm{gas}\left(\mathrm{Al},\mathrm{CO},\mathrm{Mg}\right)+3.04\times 10^{-1}{\mathrm{Al}}_{4}C_{4}\mathrm{Si}+2.90\times 10^{-1}{\mathrm{Al}}_{4}O_{4}C \end{array}$$

From Equations (2)–(4), one can expect chemical reactions at the melt/filter interface with possible formation of different carbides and the oxocarbide Al_4_O_4_C. With formation of the gas phase, no equilibrium will be established at the melt/filter interface under vacuum conditions as shown by Equations (3) and (4). According to Equation (2), the melt partially reacts with the filter material to form mainly Al_8_SiC_7_ and Al_4_O_4_C, but no gas. Therefore, it is possible that a stable and possibly protecting layer can be formed under ambient pressure conditions. The chemical stability of Al_8_SiC_7_ and Al_4_O_4_C against the aluminum melt at ambient pressure and a temperature of 740 °C was also investigated by thermodynamic calculations as follows, Equations (5) and (6):

(5) $$\begin{array}{cc} 0.5\;L\left(\mathrm{Al},\mathrm{Si},\mathrm{Mg}\right) & +0.5{\;\mathrm{Al}}_{4}O_{4}C\\ & \to 4.99\times 10^{-1}\;L\left(\mathrm{Al},\mathrm{Si},\mathrm{Mg}\right)+4.75\times 10^{-1}{\mathrm{Al}}_{4}O_{4}C+1.92\times 10^{-2}\mathrm{spinel}\\ & +6.27\times 10^{-3}{\mathrm{Al}}_{8}{\mathrm{SiC}}_{7} \end{array}$$

(6) $$0.5\;L\left(\mathrm{AL},\mathrm{Si},\mathrm{Mg}\right)+0.5{\;\mathrm{Al}}_{8}{\mathrm{SiC}}_{7}\to 0.5L\left(\mathrm{Al},\mathrm{Si},\mathrm{Mg}\right)+0.5{\mathrm{Al}}_{8}{\mathrm{SiC}}_{7}$$

As shown by Equations (5) and (6), Al_4_O_4_C and Al_8_SiC_7_ were mainly or completely stable against the melt. Based on these results, the formed interfacial layer should be relatively stable against aluminum melt at casting conditions under ambient pressure. However, at the beginning of the casting, the first interface will be a melt/filter/air interface. The results of the thermodynamic calculation for that case can be expressed by, Equation (7):

(7) $$\begin{array}{cc} 0.25\;L\left(\mathrm{Al},\mathrm{Si},\mathrm{Mg}\right) & +0.25\left(0.79\mathrm{Ar}+0.21\;O\right)+\;0.5\;\mathrm{filter}\\ & \to 4.94\times 10^{-1}{\;\mathrm{Al}}_{4}O_{4}C+2.25\times 10^{-1}{\mathrm{Al}}_{8}{\mathrm{SiC}}_{7}+1.98\times 10^{-1}\mathrm{gas}\left(\mathrm{Ar},\mathrm{Mg},\mathrm{CO}\right)\\ & +2.69\times 10^{-2}{\mathrm{Al}}_{4}C_{4}\mathrm{Si}+4.55\times 10^{-2}\mathrm{graphite}+1.19\times 10^{-2}\;\mathrm{spinel} \end{array}$$

which showed that mainly Al_4_O_4_C and Al_8_SiC_7_ will be formed at the melt front during casting in contact with air.

## 4. Discussion

This study examined the filtration behavior of Al_2_O_3_-C filter with AlSi7Mg melt. These were complemented by thermodynamic calculations and wetting measurements. The wetting behavior of Al_2_O_3_-C substrates in contact with AlSi7Mg was evaluated by means of a conventional sessile drop test (vacuum, 950 °C and 180 min) and a sessile drop test with capillary purification unit (vacuum, 730 °C and 30 min). The conventional sessile drop test yielded a contact angle of around 92°, contradicting the statement of the good slagging resistance due to poor wetting. The sessile drop measurement with capillary purification showed difficulties in measuring the contact angle due to the strong non-wetting behavior accompanied by rolling and jumping of the drop at the Al_2_O_3_-C substrate. A determination of the apparent contact angle of the rolling drop yielded a value of 157°, which is strongly non-wetting. The difference between the conventional sessile drop and the sessile drop with capillary purification explicitly proved the importance of the testing conditions for determination of the contact angle by aluminum melt. Sessile drop measurements at higher temperatures might enhance reactions not occurring within an industrial aluminum filtration setup.

Although the total level of inclusions in the aluminum melt before filtration was unavoidably different for the two industrially conducted filtration trials, there were significant differences between the filtration behavior of Al_2_O_3_-C filters and Al_2_O_3_ reference filters. For both filtration trials, the PoDFA index of the Al_2_O_3_-C filter sample was equal to half of the PoDFA index of the Al_2_O_3_ reference filter sample, indicating a significantly improved filtration when using Al_2_O_3_-C filter. Possible explanations for the improved filtration might be the reducing effect of the Al_2_O_3_-C or the strong non-wetting behavior between Al_2_O_3_-C and AlSi7Mg, Voigt et al. [3].

Notable is the observation of a newly formed layer between the aluminum and the Al_2_O_3_-C coating, whereby such a layer was not identifiable in each trial. The layer possessed thicknesses between 10 µm up to 50 µm and consisted of Al, C, and O. Additionally, thermodynamic calculations with parameters of the wetting and filtration trials were conducted and revealed the formation of an Al_4_O_4_C phase. This was in accordance with the detected Al, C, and O layers. The thermodynamically predicted phase Al_8_SiC_7_ could not be detected at the interface between the solidified aluminum melt and the Al_2_O_3_-C filters, neither by EBSD nor by EDX.

## 5. Conclusions

The wetting behavior performed by two different methods and filtration behavior of Al_2_O_3_-C filters estimated by PoDFA analysis has been measured and compared to the behavior of standard Al_2_O_3_ filters. 

Al_2_O_3_-C filters are shown to be more effective concerning the removal of non-metallic inclusions from the aluminum alloy AlSi7Mg melts when using the developed casting system with a sand mold. Additional investigations are necessary to verify the results, to understand the complex filtration mechanism of the Al_2_O_3_-C filters and distinguish between the filtration performance of the two different Al_2_O_3_-C materials coked at 800 °C and 1400 °C respectively. The surface morphology as well as phase characteristics have to be taken into account.

## Figures and Tables

**Table 1:** Composition and thermal treatment conditions for the preparation of Al_2_O_3_ coating. (* based on sum of solids)**.**

Raw Materials and Thermal Treatment Conditions	Function	Fraction/wt.%
Al_2_O_3_ CT 9 FG (Almatis, Ludwigshafen, Germany)/wt.%	Refractory material	33.3
Al_2_O_3_ CT 3000 SG (Almatis, Ludwigshafen, Germany)/wt.%	Refractory material	33.3
Al_2_O_3_ T60/T64 45µm (Almatis, Ludwigshafen, Germany)/wt.%	Refractory material	33.3
Optapix AC 170* (Zschimmer & Schwarz, Lahnstein, Germany)/wt.%	Binder	1.0
Dolapix CE 64* (Zschimmer & Schwarz, Lahnstein, Germany)/wt.%	Dispersant	0.6
Sintering temperature of the coating/°C		1600
Atmosphere		oxidizing

**Table 2:** Composition and thermal treatment conditions for the preparation of Al_2_O_3_-C coating. (* based on sum of solids).

Raw Materials and Thermal Treatment Conditions	Function	Fraction/wt.%
Al_2_O_3_ Martoxid MR-70 (Martinswerk, Bergheim, Germany)/wt.%	Refractory material	66.0
Coal pitch Carbores P (Rain Carbon, Castrop-Rauxel, Germany)/wt.%	Binder	20.0
Carbon black N991 (Lehmann & Voss & Co., Hamburg, Germany)/wt.%	Filler	6.3
Graphite AF 96-97 (Graphit Kropfmühl, Hauzenberg, Germany)/wt.%	Filler	7.7
Ammonium ligninsulfonate* (Otto Dille, Norderstedt, Germany)/wt.%	Binder + dispersant	1.5
MelPers^®^ 9360* (BASF, Ludwigshafen, Germany)/wt.%	Dispersant	0.3
Contraspum K 1012* (Zschimmer & Schwarz, Lahnstein, Germany)/wt.%	Anti-foaming agent	0.1
Coking temperature of the coating/°C		800 and 1400
Atmosphere		reducing

**Table 3:** Results of the PoDFA analysis of the filtration trial 1, regarding the filtered melt.

Inclusion Types	Al_2_O_3_ Reference	Al_2_O_3_-C 800 °C
Al_2_O_3_ films/number kg^−1^ (Length < 500 µm, thickness < 3 µm)	187	180
Carbides/mm^2^ kg^−1^	0.006	0.003
Magnesium oxide/mm^2^ kg^−1^	0.004	0.003
Spinel/mm^2^ kg^−1^	0.198	0.076
Reacted refractory material (spinel related)/mm^2^ kg^−1^	0.015	0.018
Non-reacted refractory material (α-Al_2_O_3_, CaO, SiO_2_)/mm^2^ kg^−1^	0.009	0.002
Iron and manganese oxides/mm^2^ kg^−1^	0.005	0.003
Grain refiner TiB_2_/mm^2^ kg^−1^	0.009	0.001
PoDFA index (sum)/mm^2^ kg^−1^	0.246	0.106

**Table 4:** Results of the PoDFA analysis of filtration trial 2, regarding the filtered melt.

Inclusion Types	Al_2_O_3_ Reference Rough	Al_2_O_3_-C 800 °C	Al_2_O_3_-C 1400 °C
Al_2_O_3_ films/number kg^−1^ (Length < 500 µm, thickness < 3 µm)	66	65	106
Carbides/mm^2^ kg^−1^		0.019	
Magnesium oxide/mm^2^ kg^−1^	0.055		0.012
Spinel/mm^2^ kg^−1^	0.076	0.029	0.070
Reacted refractory material (spinel related)/mm^2^ kg^−1^	0.765	0.395	0.492
Non-reacted refractory material (α-Al_2_O_3_, CaO, SiO_2_)/mm^2^ kg^−1^	0.043	0.039	0.019
Non-reacted refractory material (Graphite)/mm^2^ kg^−1^	0.12		
Iron and manganese oxides/mm^2^ kg^−1^			0.025
PoDFA index (sum)/mm^2^ kg^−1^	1.06	0.482	0.618

## References

[1] Olson R.A., Martins L.C.B. (2005). Cellular ceramics in metal filtration. Adv. Eng. Mater..

[2] Kondrat’Ev A.S., Popov V.N., Aksel’Rod L.M., Baranovskii M.R., Suvorov S.A., Tebuev N.B. (1990). Possibilities of filter-refining of metals and the required characteristics of the filter elements: A review. Refract. Ind. Ceram..

[3] Voigt C., Ditscherlein L., Werzner E., Zienert T., Nowak R., Peuker U., Sobczak N., Aneziris C.G. (2018). Wettability of AlSi7Mg alloy on alumina, spinel, mullite and rutile and its influence on the aluminum melt filtration efficiency. Mater. Des..

[4] Routschka G., Wuthnow H. (2012). Handbook of Refractory Materials.

[5] Emmel M., Aneziris C.G. (2012). Development of novel carbon bonded filter compositions for steel melt filtration. Ceram. Int..

[6] Luchini B., Hubálková J., Wetzig T., Grabenhorst J., Fruhstorfer J., Pandolfelli V., Aneziris C. (2018). Carbon-bonded alumina foam filters produced by centrifugation: A route towards improved homogeneity. Ceram. Int..

[7] Eustathopoulos N., Nicholas M.G., Drevet B. (1999). Wettability at High Temperatures.

[8] Bao S., Tang K., Kvithyld A., Tangstad M., Engh T.A. (2011). Wettability of aluminum on alumina. Met. Mater. Trans. B.

[9] Sobczak N., Singh M., Asthana R. (2005). High-temperature wettability measurements in metal/ceramic systems—Some methodological issues. Curr. Opin. Solid State Mater. Sci..

[10] Shen P., Fujii H., Matsumoto T., Nogi K. (2004). Critical factors affecting the wettability of α-alumina by molten aluminum. J. Am. Ceram. Soc..

[11] Fankhänel B., Stelter M., Voigt C., Aneziris C.G. (2017). Interaction of AlSi7Mg with oxide ceramics. Adv. Eng. Mater..

[12] Damoah L., Zhang L. (2010). Removal of inclusions from aluminum through filtration. Met. Mater. Trans. B.

[13] Görner H., Syvertsen M., Øvrelid E.J., Engh T.A. (2005). AlF3 as an aluminium filter medium. Light Metals.

[14] Zhou M., Shu D., Li K., Zhang W.Y., Ni H.J., Sun B.D., Wang J. (2003). Deep filtration of molten aluminum using ceramic foam filters and ceramic particles with active coatings. Met. Mater. Trans. B.

[15] Luyten J., Vandermeulen W., De Schutter F., Simensen C., Ryckeboer M. (2006). Ceramic foams for Al-recycling. Adv. Eng. Mater..

[16] Syvertsen M., Kvithyld A., Bao S., Nordmark A., Johansson A. (2014). Parallel laboratory and industrial scale aluminium filtration tests with Al_2_O_3_ and SiC based CFF filters. Light Metals.

[17] Voigt C., Jäckel E., Taina F., Zienert T., Salomon A., Wolf G., Aneziris C.G., Le Brun P. (2017). Filtration efficiency of functionalized ceramic foam filters for aluminum melt filtration. Met. Mater. Trans. B.

[18] Voigt C., Dietrich B., Badowski M., Gorshunova M., Wolf G., Aneziris C.G. (2019). Impact of the filter roughness on the filtration efficiency for aluminum melt filtration. Light Metals.

[19] Utigard T.A., Sommerville I. (2005). Cleanliness of aluminum and steel: A comparison of assessment methods. Light Metals.

[20] Canullo M., Labaton M.F.J., Laje R.A. Cleanliness of primary A356 alloy: Interpretation and standardisation of PODFA laboratory measurements. Proceedings of the Aluminum Cast House Technology: 8th Australasian Conference TMS.

[21] Bianconi A., Bachrach R.Z., Hagstrom S.B.M., Flodström S.A. (1979). Al- Al2O3 interface study using surface soft-x-ray absorption and photoemission spectroscopy. Phys. Rev. B.

[22] Sobczak N., Asthana R., Radziwill W., Nowak R., Kudyba A. (2007). The role of aluminum oxidation in the wetting-bonding relationship of Al/oxide couples. Arch. Metall. Mater..

[23] Andersson J.O., Helander T., Höglund L., Shi P., Sundman B. (2002). Thermo-Calc & DICTRA, computational tools for materials science. Calphad.

[24] Salomon A., Zienert T., Voigt C., Jäckel E., Fabrichnaya O., Rafaja D. (2013). Comparison of interfacial reactions between AlSi7Mg and alumina filter after casting and spark plasma sintering**. Adv. Eng. Mater..

[25] Zienert T., Fabrichnaya O. (2013). Phase relations in the A356 alloy: Experimental study and thermodynamic calculations. Adv. Eng. Mater..

[26] Gröbner J., Lukas H.L., Aldinger F. (1996). Thermodynamic calculation of the ternary system Al-Si-C. Calphad.

